# Microeconomic forecasting: Constructing commensurable futures of educational reforms

**DOI:** 10.1177/0306312719837364

**Published:** 2019-03-18

**Authors:** Guus Dix

**Affiliations:** Max-Planck-Institut für Gesellschaftsforschung, Köln, Germany; Center for Science and Technology Studies – Science and Evaluation Studies, Leiden University, Leiden, The Netherlands

**Keywords:** boundary organizations, commensuration, economic expertise, economic policy, forecasts

## Abstract

According to economists from the Netherlands Bureau for Economic Policy Analysis, the introduction of performance pay for primary and secondary school teachers would lead to an increase in Dutch GDP of one-and-a-half percent in 2070. A new epistemic practice of microeconomic forecasting undergirded this attempt to make the distant future part of the political present. Taking the construction of the economic growth potential of performance pay as a starting point, this article analyzes how microeconomic forecasting emerged in one of the world’s oldest forecasting bureaus – and to what consequences. First, it highlights the institutional preconditions for this ‘turn to micro’ in an institution that had pioneered in the field of macroeconomic forecasting. Second, the article analyzes microeconomic forecasting as a distinct epistemic practice that brings different forms of economic expertise together to make the future of educational reforms commensurable. Finally, it analyzes the political consequences of this new epistemic practice in the sense that it not only enables but simultaneously limits the provision of policy-relevant evidence. Beyond the specificities of the case, the article contributes to the sociological study of economic policy devices against the background of a predominant market bias in the STS research on economics.

## Introduction

In 2010, a new coalition government of Christian Democrats and Conservative Liberals (VVD) announced that there would be ‘more room for performance pay, for both individuals and teams’ in Dutch education (Rijksoverheid, 2010a: 31). Justifying the choice for performance-related bonuses for teachers, the government claimed that it would not only enhance student achievements but also contribute to the ‘Dutch ambitions for economic growth’ (Ministry of Education, Culture and Science, 2011a, 2011b: 1). The economic justification for educational reforms did not come out of thin air; it was provided by an institution that has offered economic advice to Dutch policymakers for over seventy years:

A recent calculation of the Netherlands Bureau for Economic Policy Analysis (CPB) demonstrates that the educational policy measures of the government contribute to the creation of jobs. Educational policies lead to a higher level of education on the longer run and thereby to an upward effect on employment opportunities, because people who are higher educated participate more on the labor market. (Rijksoverheid, 2010b: 14)

The calculations by CPB economists reappeared in the political debates that followed suit. The conservative-liberal prime minister, for one, said it was of the ‘utmost importance that the quality of education is at such a level that … our economy provides an environment in which commercial activities can emerge and flourish’. Again, the Bureau’s calculations stood out:

By now, the government has been busy for half a year. When I look at the report of the CPB that appeared yesterday [Van Elk et al., 2011a], I can conclude that all recommendations of the CPB are in fact implemented by the government. I refer to the reinforcement of the position of the teacher here (Tweede kamer, 2011: 1).

In a debate with opposition parties, the financial spokesperson of the VVD also defended the choice for performance pay by referring to the Bureau:

By the way, the calculations of the CPB are on my desk. Mister Pechtold [party chairman of the social-liberal party (D66)] should feel free to borrow them. In these calculations one can find an overview of the effects on educational achievements, on the quality of education, of the money that is spend. Indeed, it matters enormously how you spend the money. That’s why I think it is so sensible that the government chooses not to spend money randomly, but to take it from places where it is not well-spend and to choose to introduce performance pay. (Tweede kamer, 2010a: 24).

Finally, the VVD State Secretary of Education, who was directly responsible for its implementation, responded to critical questions from political adversaries by pointing to studies from the CPB that had demonstrated the existence of ‘evidence-based evidence (sic) for performance pay’ (Tweede kamer, 2010b: 73).

On closer inspection, the economic evidence referred to in policy documents and political debates was of very recent origin. Just before the 2010 elections, CPB economists had brought performance pay to the fore as the most promising policy measure to reform Dutch education. The introduction of bonuses for primary and secondary school teachers – based on their ability to increase student test scores – would directly enhance educational achievements and boost economic growth in the longer run. The CPB claimed that the government could expect a rise in GDP of one-and-a-half percent in 2070 if it introduced the policy measure immediately. With regard to the long-term performance of the Dutch economy, the introduction of performance pay outperformed all other policy measures that CPB economists took into consideration (CPB and PBL, 2010; Van Elk et al., 2011a, 2011b). The comparison between policy measures on the basis of their potential to induce economic growth was made possible by a brand-new epistemic practice in which microeconomic studies – not macroeconomic indicators – played a pivotal role. Taking the construction of the economic growth potential of performance pay as a starting point, I analyze how this practice of microeconomic forecasting emerged in one of the world’s foremost forecasting bureaus – and with what consequences.

With its focus on economic science and its wider ramifications, my article is part of a flourishing body of Science and Technology Studies (STS) research on the role of economic models and theories in contemporary life (Callon, 1998; Callon et al., 2007; MacKenzie et al., 2007; Pinch and Swedberg, 2008). Reviewing these engagements with economics, however, Hirschman and Berman (2014) discern a clear market-bias among STS scholars, in the sense that they ‘have looked at how economists’ devices affect markets rather than policy’ (p. 782). For a more balanced account, they say, the sociological analysis of market devices should be complemented by an analysis of ‘economic policy devices’ defined as ‘the wide variety of sociotechnical tools that help policymakers see and make decisions about the world in economic ways’. This balance is to be particularly welcomed in the case of economic forecasts because ‘they are a key source of information for economic policy-makers and for the legitimation of political decisions’ (Beckert, 2016: 239). Siding with recent studies on the political role of microeconomics and econometrics (Hood, 2017; Williams and Cook, 2016), this article draws on the case of microeconomic forecasting to make clear what an STS-inspired analysis of economic policy devices amounts to. Three requirements stand out here.

First, an STS-inspired analysis should consider the *institutional preconditions* for the political involvement of economists and their devices. To capture the preconditions for microeconomic forecasting, it is fruitful to analyze the CPB as a quintessential boundary organization, its having mediated between the worlds of economic science and Dutch politics since its establishment (De Vries et al., 2010; Guston, 1999; Halffman, 2009). Second, a sociological analysis of economic policy devices should study them as particular *epistemic practices*. Drawing on the literature on commensuration and comparability (Espeland and Stevens, 1998, 2008; Schinkel, 2016), I analyze microeconomic forecasting as a model-based and interpretative endeavor which makes the future of wide variety of interventions commensurable by combining different kinds of economic expertise. Finally, a sociological analysis of economic policy devices should pay attention to their *political consequences* in so far as these consequences are bound up with the epistemic practices and institutional preconditions in question. I show how microeconomic forecasting not just enabled but simultaneously constrained the provision of policy-relevant evidence (Breslau, 2013; Espeland and Stevens, 1998; Parkhurst, 2017).

## Microeconomic forecasting: Boundary organizations, commensurable futures and the loss of contextual information

The concern with the future goes a long way back in economics. When economists talk about that future they usually have the aggregate economy in mind, and existing studies in STS hence focus on the construction and use of macroeconomic models that operate at this level. In a range of studies, historians and sociologists of science have explored how economists began to chart economic trends and tendencies in the early decades of the twentieth century to predict what the future of the economy would look like (Friedman, 2014; Van den Bogaard, 1999a). For the period after the Second World War, they traced the shift from predicting trends to the development of mathematical models with which the future consequences of present-day changes in the economy – policy-driven or otherwise – could be calculated. These macroeconomic models came to occupy a central position in newly established forecasting bureaus and became engrained in economic policymaking in range of countries and firms (Evans, 1997, 2007; Reichmann, 2013; Van den Bogaard, 1999b). In their respective accounts of macroeconomic forecasting, these scholars converged on the idea that forecasts never directly follow from the economist’s model. Instead, the model-based extrapolations to the future become possible – and authoritative – due to the collaborative work and interpretative flexibility of the forecasters themselves as well as the epistemic participation of audiences outside the forecasting community (De Vries et al., 2010; Evans, 1997: 397, 2007: 688–690; Reichmann, 2013).

My analysis is congruent in with this key insight. But although microeconomic forecasting similarly requires collaboration and judgment, it also differs significantly from macroeconomic forecasting in terms of its timeframe and epistemic practice. Macroeconomic forecasts, for one, primarily extrapolate to the state of the economy in the short- and medium-term. To make that extrapolation possible, macroeconomic models work with a large number of mathematical equations that express the relationship between different economic variables such as household expenditures, rate of interest, private and public indebtedness, government spending and international trade. Microeconomic forecasting, on the contrary, seeks to bypass the volatility bound up with short- and medium-term economic developments. To extrapolate to the very distant future, it leaves out all these economic variables and only models the relationship between specific policy interventions and human-capital driven economic growth. Because the scholarship on macroeconomic forecasting offers little additional guidance, I draw on other strands of STS to address three related questions regarding the institutional preconditions, the epistemic practice and the political consequences of microeconomic forecasting.

The first question concerns the institutional preconditions for the development of economic policy devices: What made microeconomic forecasting possible in an institution that had derived its authority from macroeconomic forecasting for over sixty years? To understand the preconditions for this new epistemic practice, I analyze the CPB as a quintessential boundary organization (Guston, 1999). A boundary organization, as Guston (1999) defines it, is an organization that ‘exists on the frontier of two relatively distinct social worlds with definite lines of responsibility and accountability to each’ (p. 93). As such, it creates a space in which different kinds of actors – such as economic experts and policymakers – interact and creates objects – such as the economic future – to which these actors can relate in their own specific way. In the case considered here, the Bureau’s position on the frontier of the social worlds of politics and academic economics is key to understanding its ‘turn to micro’ (De Vries et al., 2010; Halffman, 2009). In terms of the political responsibility of the CPB, the emergence of microeconomic forecasting was conditional on the major privatization and marketization operations of the Dutch government in the 1990s. With welfare state reforms becoming a key issue in political debates, the Bureau turned to microeconomic expertise in order to remain policy-relevant. In terms of the CPB’s scientific responsibility, microeconomic forecasting was conditional on the rise of the idea of experimentation as the mark of scientific rigor in economics (Bowles, 2016; Levitt and List, 2009; List, 2008, 2011; Panhans and Singleton, 2017). At the intersection of these two responsibilities, a group of CPB economists became convinced that experimental studies were a potent source for recommending or discrediting educational reforms.

The second question concerns the analysis of an economic policy device as a particular epistemic practice: How should we understand microeconomic forecasting as a practice of producing knowledge about the economic future? The notion of practice is understood here as an array of human activities in which habitual and creative behavior intersect. As such, epistemic practices depend on shared scientific skills and understandings but equally presuppose the ability to actively and creatively respond to new challenges – such as new scientific and political demands in this particular case (Pickering, 1992: 3; Schatzki, 2001: 2–4). To address the question of microeconomic forecasting as an epistemic practice, I draw on the literature on commensuration and comparability (Espeland and Stevens, 1998, 2008; Schinkel, 2016). As a social process, commensuration entails the active construction of a particular dimension that anchors the comparison between different objects. These objects are made sufficiently similar that they can be grouped together but have to remain sufficiently different to be ordered hierarchically. Commensuration is hence part of a broader desire to make objects comparable by ‘stabilizing a space in which these objects, along with potential others, can occur alongside each other in the first place’ (Schinkel, 2016: 377). In many areas of human activity, this desire for comparability is bound up with a moral-epistemic ideal that combines the ‘objective’ assessment of actors or institutions with the attempt to make them responsible for enhancing their own performance (Espeland and Sauder, 2007; Fourcade, 2016; Sauder and Espeland, 2009). In the second part of the article, I argue that to understand microeconomic forecasting as an epistemic practice we should see it as attempting to construct commensurable futures. To make the future of educational reforms commensurable – and make these reforms comparable – economists from the Bureau engaged in four related activities. They first stabilized the yield of foreign economic experiments so as to quantify the expected increase of the performance of Dutch students. Second, they built a model that could trace the effects of that increased performance on the school trajectory of different cohorts of students. Third, they translated the increased time Dutch students would spend in school and the future composition and productivity of the Dutch labor force into a long-term GDP score for every policy measure. Finally, they calculated the budget that was required to secure the full economic growth potential of each individual policy measure.

The third question has to do with the political consequences of economic policy devices: In what way does microeconomic forecasting not just enable but simultaneously constrain the provision of policy-relevant evidence? A prevalent argument in the sociology of macroeconomic forecasting is that forecasts can be politically useful even when they are wrong. That is, although economic forecasts are often (highly) inaccurate, the political involvement of forecasters might be valuable for modern democratic societies because they form an expert community that maintains a form of life in which scientific values predominate (Evans, 1997: 397, 2014: 249–250). The epistemic participation of economic and political actors in the wider social network of the forecasting community would foster a more open discussion about the economic and moral presuppositions of different scenarios (Evans, 1999: 354, 2007; Reichmann, 2013). In addition, the political institutionalization of economic forecasting makes it a coordinating device that helps to reach consensus in a chaotic environment, while providing an element of openness and surprise at the very same time (Beckert, 2016: 231–237). Though the emphasis on participation, coordination and consensus is valuable in itself, these studies pay remarkably little attention to the ways in which economic evidence limits the values and perspective on the future that actors might bring into play. As Parkhurst (2017) argues, a politically informed account of evidence-based policymaking should start ‘from a recognition that policies typically involve multiple social concerns, and there can be different evidence bases relevant to each of them’ (p. 6). The third part of this article extends upon this idea and shows that commensuration and economic framing make certain concerns invisible or irrelevant while increasing the visibility and relevance of others (Breslau, 2013; Espeland and Stevens, 1998). The wider variety of knowledge claims in economics (epistemic diversity) and the specific characteristics of the Dutch educational system (institutional feasibility) are part of the context that is lost in commensuration.

### Methods

The article builds on document analysis and expert interviews. For the first two sections of the article – those on the turn to micro and the construction of commensurable futures – I have analyzed a series of published CPB reports, working papers and internal and external evaluations of the Bureau in the period between 2001 and 2011. These documents were concerned with the microeconomics of public sector reform and with experiments on performance pay in education. With the historical scholarship on the CPB as an additional source of information, they enabled me to trace the shift to microeconomic expertise at the Bureau and present a first outline of microeconomic forecasting as an epistemic practice. Against the background of that preliminary document analysis, I conducted four expert interviews with (former) CPB economists working on microeconomic forecasting or on previous projects having to do with the economics of education.^1^ These interviews enabled me to generate a more in-depth understanding of (1) the Bureau’s position on the frontier of politics and academic economics; of (2) the role of collaboration, interpretation and judgment in producing knowledge about the future; and of (3) the underlying objective of making educational reforms comparable. For the third part of the article, I conducted eight interviews with experts who were involved in the implementation phase of performance pay: four academic economists and one academic sociologist with expertise in the economics of education and experimental economics, two civil servants of the Ministry of Education, Culture and Science responsible for the implementation of performance pay, and a representative of the union who fought against the policy measure. These interviews enabled me to gain (4) a more in-depth understanding of the considerations concerning the epistemic diversity and institutional feasibility that were lost in the process of making futures commensurable – considerations that cannot be extracted from any of the published expert documents on microeconomic forecasting or policy documents on performance pay in education.

## The turn to micro: The institutional preconditions for microeconomic forecasting

From its inception, the Netherlands Bureau for Economic Policy Analysis has been indebted to the field of academic economics for new recruits and input for (and feedback on) its epistemic practices as well as to the political field for orientation for its future research projects and for the resources to support them. The CPB was established immediately after the Second World War to advise the Dutch government in matters of macroeconomic policy. As a new advisory institute, the economic future became the Bureau’s state-sanctioned core concern. To make that possible, the CPB was a split-off from the Central Bureau of Statistics (CBS), with an institutional division of labor in which the former would make predictions on the basis of data about the past gathered by the latter. The CPB obtained a formal status by law as part of the Ministry of Economic Affairs in 1947 but it could operate without direct government interference in practice.

Though it operated at a certain distance from the political arena, the Bureau’s macroeconomic forecasting practices were nevertheless bound up with postwar social and political divisions in the Netherlands (Van den Bogaard, 1999b: 346–348). Both before and immediately after the war, Dutch society was strongly divided between liberal, socialist, catholic and protestant groups and their political representatives – so-called ‘pillars’. After attempts to reduce the political importance of these pillars had failed in the early post-war period, a particular conception of macroeconomic planning had to be found that was acceptable to all of them. The econometric model developed by Jan Tinbergen stood at the heart of that conception. Tinbergen’s model consisted of a set of mathematical equations – a ‘machine’ or ‘automaton’ in his words – that captured important structural features of the economy. As a closed system of causal relations, this econometric model enabled economists to calculate how current changes in the economic parameters – due to policy changes, for instance – would affect the future course of the Dutch economy. In a highly fractured political arena, Tinbergen’s model came to be looked upon favorably because it could contribute to the establishment of a coherent national policy. With its mathematical formalism as sign of scientific neutrality – instead of ideological position-taking – the Bureau’s macroeconomic forecasting practice helped politicians to reach a consensus on economic policymaking without harming the existing pillared identities (Van den Bogaard, 1999a: 300–305, 1999b; Schuyt and Taverne, 2004).

The macroeconomic forecasts of the CPB remain central to the Bureau’s epistemic and political authority to this day, and play an important role in processes of policy preparation and legitimation – even after severe professional criticism of their underlying behavioral assumptions and after a public controversy, following the recent financial crisis, over their lack of predictive accuracy (Butter, 2011; Don and Verbruggen, 2006; Halffman and Hoppe, 2005).^2^ Against the background of this long tradition of macroeconomic forecasting, the interest in microeconomics is a relatively recent one. In the late 1990s and early 2000s, the Bureau began to hire economists with a stronger background in the economics of the public sector. The Bureau’s partial ‘turn to micro’ was an important institutional precondition for the development of microeconomic forecasting as a new economic policy device. Existing at the boundary of the worlds of politics and science, two external developments come together here. On the one hand, microeconomic forecasting was conditional on the Bureau’s attempt to remain policy-relevant and authoritative at a time when the political debate shifted significantly. On the other hand, microeconomic forecasting was conditional on the Bureau’s attempt to attune to the rise of the demand for solid experimental support for scientific claims in academic economics.

### Attuning to political developments: Welfare state reforms and the policy-relevance of economic expertise

The external scientific committee that reviewed the Bureau in 2003 judged that ‘the CPB has successfully maintained its reputation’ so far, but also noted that ‘the economic policy debate has broadened from macroeconomic management to issues like regulation and welfare reform’ (CPB Review Committee, 2003: 7, 15). Although the committee appreciated the fact that the CPB had already begun to shift to ‘the new challenging fields of structural reforms and microeconomic analysis’ it should further ‘strengthen its position … by broadening its research questions and research methods to issues like welfare reform and regulation’ (CPB Review Committee, 2003: 9, 33). The CPB (2003: 26, 62–66) responded to the review committee by saying it equally valued its new-found expertise on these issues but that the transfer of even more resources to microeconomic studies would endanger the ability to continue with its macroeconomic forecasting practices.^3^ Seeking to keep up with current policy debates with limited resources, the Bureau initially put ‘incentives’ at the center of its microeconomic research agenda.

Two economists who arrived at the CPB in the 1990s recalled a significant political interest in the issue of incentives in the public sector. Between 1994 and 2002, two ‘purple’ cabinets of conservative liberals (VVD), social democrats (PvdA) and social liberals (D66) had started ‘a wave of privatization … a wave totally preoccupied with market ordering’ (CPB economist 4). The interest in incentives on the part of the ministries and the government should be understood in light of that wave of welfare state reform:

Then there was suddenly a great interest in incentives. This can also be related to the time, it was around the year 2000, that there was a great interest from the ministries. And then there was a lot of thinking about: How can we force all kinds of sectors to open up? (CPB economist 3)

These attempts to introduce market forces through privatizing state institutions and fostering competition started with heavily regulated sectors such as taxi driving, notaries and real estate. Subsequently, the attention shifted towards so-called ‘network sectors’ such as the provision of water and energy and the systems of public transport. Whatever their specific institutional features, though, the attempts to forcefully open up these sectors was bound up with the question of incentives:

The next logical step was: We want the incentives to be right everywhere. It was very much commonplace to think like that in those days. How is government doing in that regard? And with ‘government’ you have to immediately admit that the biggest part, in fact a very big part, is placed at a distance from the state. (CPB economist 4)

On the basis of their respective areas of expertise, CPB economists began to explore the incentive structure of organizations that the government had placed at a distance – unemployment agencies, police departments, research and development initiatives, and academic institutions (Cornet and Rensman, 2001; Koning and Deelen, 2003; Pomp et al., 2003; Vollaard, 2003). Monetary incentives were at the heart of performance pay too. Hence the idea to introduce bonuses for teachers made its first appearance at the Bureau in the context of this broader project on ‘incentives for semi-public services’ (CPB, 2001: 134–136, 2003: 15; see also Canton and Webbink, 2004: 17; Koning et al., 2004).

### Attuning to scientific developments: Academic economics and the demand for solid experimental support

As a boundary organization, the CPB does not respond only to developments in the political field; it is equally responsive to scientific developments. In the case considered here, the development of microeconomic forecasting as a new epistemic practice was made possible by a recent turn to experiments in academic economics. Dissecting the cognitive culture of modern economics in the late 1990s, Breslau and Yonay (1999) could still write that ‘economics is hardly an experimental science and does not depend on the mediation of laboratory apparatus’ (p. 318). Twenty years later, the laboratory has become much more prominent in the production of economic knowledge (Bowles, 2016; Guala, 2005: 13–38). Of even greater importance to microeconomic forecasting is the rise of field experiments. Although one can point to predecessors in social scientific experimentation – from agricultural experiments in the 1920s to government-initiated social experiments in the 1960s – the veritable ‘surge of field experiments in economics’ dates from in the late 1990s (Levitt and List, 2009: 2). In agreement with laboratory-based economic experimenters, proponents of field experiments equally hold that data should be generated, rather than collected, and that economic science would do well to move from model-building to empirical methods. In contrast to their colleagues, however, field-based economic experimenters argue that the random distribution of individuals over control and treatment groups should take place in a natural environment – not under artificial laboratory constraints (List, 2008: 204–206, 2011: 5; Panhans and Singleton, 2017).

Academic economists’ pleas for experiments left their mark on the Bureau. Although CPB economists did not begin to conduct field experiments, they embraced the idea of experimentation as the mark of scientific rigor: ‘When you look at the Bureau, certainly at the time, there was a very strict school who considered that everything had to be experimental evidence’ (Academic economist 2). CPB economists relate that newfound strictness to the rise of experimentation as a new standard: ‘there are more and more scientists that say: this is the golden standard, this is what you have to aim for. So it’s a wave that is not specific to the CPB’ (CPB economist 4). That experimental wave was particularly pronounced in the sub-discipline of economics at the heart of the policy measures considered here: ‘out of the economics of education emerged a culture that we only believe results if there is solid support, so with experimental variation in it. So, we do not like correlations, associations’ (CPB economist 3). The opposition between ‘experimental variation’, on the one hand, and ‘correlations’ and ‘associations’, on the other, is mirrored in the assessment of scientific experts on education with a different disciplinary background: ‘I think that sociologists are sometimes somewhat more flexible with their methods so we might have different points of view on that score’ in the sense that they say things such as, ‘coming from the educational field there are also surveys that show that …’ (CPB economist 1).^4^

When the CPB began to assess the available evidence in the economics of education, it first came across existing literature reviews that dealt with the relationship between the input of education (the money invested in salaries, buildings and ICT) and its output (student performance). The overall picture of input-output relations in these reviews was blurry and many of the effects discussed in literature were found to be statistically insignificant on closer inspection. Against the background of that initial disappointment, CPB economists decided to opt for a new kind of policy-oriented report in which they only selected ‘studies with an experimental set-up’ (CPB economist 3). On the basis of that delimitation, they could review the effects of educational reforms – i.e. economic experiments – on student performance:

What we had in mind with *Promising knowledge policies* was to make a sort of overview: What do we know on the basis of the economics of education in terms of the policy measures that work very well and what does not work well? (CPB economist 3)

Initially, there was little calculation involved, as policy measures were simply granted a ‘plus’, a ‘neutral’ or a ‘minus’ on basis of the available studies. There were not that many experiments at the time: ‘I think we had only a few studies on performance pay in education’ (CPB economist 3). On the basis of these studies, the use of monetary incentives for teachers figured prominently in two of the three policy measures in primary and secondary education that were granted a ‘plus’ – enhancing the quality of teaching and reducing the number of school drop-outs (the third one being the investment in pre-school education for disadvantaged students). Other interventions were considered to be neutral (subsidizing adult education) or received a ‘minus’ (diminishing class size) (cf. Cornet et al., 2006).

### From a qualitative assessment to a quantified future

Although the CPB is not primarily engaged in scientific debates nor directly involved in political struggles, it is responsive to developments in economics and politics. As a boundary organization, its partial turn to microeconomics should be understood in light of the continuing political interest in welfare state reforms and the increased interest of academic economists in field experiments (De Vries et al., 2010; Guston, 1999; Halffman, 2009). The qualitative assessment of educational reforms was the first full-fledged result at the intersection of these two developments. It was presented to Dutch policymakers with the purpose of giving a kind of expert opinion: ‘I do remember that *Promising knowledge policies* – this was in 2006 – … landed well in policy circles. They were really waiting for a convenient booklet with the pros and cons of policy measures’ (CPB economist 3).

Although the report landed well, it did little to ameliorate a protracted political concern about the Bureau’s focus on the short-term effects of reforms. Already in 2002, the trade union and the employers’ organization complained, in a debate over disability insurance reform, that the CPB neglected potential positive effects over the long run. In the absence of ways to represent the distant future, all ambitions of the government beyond its four years in office were thwarted by the emphasis on current expenses. The issue reached a climax in a major consultative body, the Social and Economic Council: ‘then someone from the employers’ organization said, like, yes, if it was up to the CPB, the ships would still be in [front of] the Normandy port’ (CPB economist 4).^5^ What was considered problematic by employers was also a matter of concern to political parties. Like earlier epistemic choices, the choice to quantify the long-term consequences of welfare state reforms is closely bound up with a political demand.

This basically sprang from a need from politics: that investments in education or trees did not make a difference. They only cost money in the models of the CPB and there was no direct benefit. … And this satisfied a political need, from political parties, who frequently complained: ‘we don’t see any return, it only costs us money’. (CPB economist 2)

Building on their initial success, economists from the Bureau expressed the need to go beyond the qualitative assessment of experimental evidence in the direction of a quantified future. They singled out the endeavor to estimate the effects of individual policy measures on labor productivity as ‘the main challenge for macro model building at CPB in the coming years’ (Don and Verbruggen, 2006: 165).

## Constructing commensurable futures: Microeconomic forecasting as an epistemic practice

Microeconomic forecasting was an effort to put the Bureau’s recent turn to micro to new ends. The challenge was substantial: to bridge the gap between experimental studies dispersed across the globe and the future productivity of the Dutch labor force. To extrapolate from the one to the other, CPB economists developed a new epistemic practice that creatively linked the recent interest in experimental studies to the Bureau’s longstanding model-building expertise. The endeavor to construct commensurable futures best captures microeconomic forecasting as an epistemic practice. To make the future of different educational reforms commensurable, CPB economists had to create one particular dimension – the economic growth potential – that made these reforms sufficiently similar to be compared with one another and sufficiently different to be ordered hierarchically (Espeland and Stevens, 1998, 2008; Schinkel, 2016). The extrapolation to the distant economic future that anchored the comparison between policy-measures could not be derived from the existing literature nor from a model that was already well-established. As was the case with macroeconomic forecasting (Evans, 1997: 397, 2007: 688–690), the construction of the economic growth potential of educational reforms required the combination of different forms of expertise – notably in microeconomics and model-building – as well as judgment from the side of the economic forecasters. Four distinct activities stand out here.

### Stabilizing the yield of policy measures

The first attempt to quantify the economic effects of policy measures was unsuccessful. Initially, the task to develop a model for returns on educational investments had been assigned to model-builders at the Bureau: ‘for quite a while they tried to tinker with a model that was somewhat more theoretical and abstract … It was more of a theoretically underpinned model they tried to calibrate empirically.’ That model did not show sufficient progress especially taking into account that ‘we were on the eve of the new elections and so it was important that we came up with a final product’. Economists from the education unit stepped in and proposed to make a simpler model that could be built in the limited time that was left: ‘then there were early elections too, but the model was just finished … I think we had just finished the overview of what policy measures would yield’ (CPB economist 2).

The second, more successful, attempt to establish a link between individual policy measures and economic growth brought the available model-building expertise and the microeconomic expertise on education closer together. In the education unit ‘we had some people that were really versed in micro-econometrics’ who were tasked to study different policy measures in more depth (CPB economist 1):

And with a number of colleagues we especially plunged in the literature to find out, at the level of policy measures, what do we know in terms of evidence about the yield of policy measures? What does the reduction of class size yield? What does the investment in the quality of teachers yield? What does additional coaching yield when it comes to reducing the number of dropouts? (CPB economist 2)

Similar to the qualitative assessment of policy measures, the microeconomists restricted themselves to experimental studies to determine the yield of a particular intervention: ‘of course we had the criterion that it must be good studies, good effect studies’ (CPB economist 2).

To create comparability, it was necessary to strike an average of the effects found in foreign experiments for each individual policy measure. Striking that average was no mechanical procedure but required judgment. First, CPB economists had to judge the value of each result, as these experiments differed in terms of the size of the control group, their time frame and the possibility to single out the actual effect of the intervention:

So certain studies also get somewhat more weight in the end result. So you look at the implementation, you look at weight, the quality that is, and then you arrive in principle at a mean effect, with those additional factors that weigh in that I just talked about, these determine what kind of effect you put on a policy measure like performance pay. (CPB economist 1)

That mean effect of foreign experiments, however, could not be directly translated into the Dutch context. In addition, there were many sources of uncertainty in the foreign experiments themselves. It proved difficult, for instance, to measure the educational gain brought about by a single teacher or team in a clear and consistent way. Moreover, the increase in test scores could be the result of teachers who focused exclusively on preparing their students for the test or by deliberate manipulation of the test scores by keeping some underperforming students out. Third, it was impossible to assess the permanency of the effects because these experiments often did not run their proper course. Finally, it was unknown whether the introduction of extrinsic rewards would harm the intrinsic motivation of teachers or whether performance pay would strengthen the divide between good and bad schools (Canton and Webbink, 2004: 22–23; CPB, 2001: 129–131; Koning et al., 2004: 94; Van Elk et al., 2011b: 44, 62; Webbink et al., 2009: 50–53).

The policy measures addressed by CPB economist could only be quantitatively compared with one another when these uncertainties were quantified too. This required a second instance of judgment. Under the heading of an ‘uncertainty variant’, CPB economists could decide that the ‘effect that is found in the foreign studies is diminished somewhat’ when they had reason to believe that the Dutch situation was somehow different from the original experimental settings (CPB economist 2). This was the case with performance pay:

For the Dutch uncertainty variant we take the risks into account that are bound up with the introduction of performance pay, such as the manipulation of educational performance by excluding weak students from the test and teaching-to-the-test. On the basis of such considerations, we cut the mean effect in half… (Van Elk et al., 2011a: 63)

The risks and uncertainties associated with the policy measure were the main reason to opt for a Dutch uncertainty variant. That choice for the uncertainty variant over the mean effect that followed directly from the set of foreign experiments is important in that it helps to stabilize the yield. Doing so enabled CPB economists to treat the formerly uncertain and risky foreign experimental results as a relatively stable relation between monetary incentives and increased student performance in the Netherlands. And only a stable mean effect can serve as the input for the model-based extrapolations to the future that adds to the comparability of policy measures (Canton et al., 2005: 33–40; Van Elk et al., 2011a: 62–65, 2011b: 34–45).

### Building a model to follow student flows

In the second activity that characterizes microeconomic forecasting as an epistemic practice, CPB economists embedded the stabilized mean effects into a stylized representation of the Dutch educational system. More specifically, the team looked for a model that could translate the yield of current educational interventions into definite shifts in the Dutch student population. The CPB has a wide range of models for extrapolating to the future at its disposal. None of the existing models, however, were deemed particularly useful for the present purpose because they ‘are very macro’ and ‘we did not need to adjust a big model in which, say, taxation, unemployment benefits and everything starts to run in tandem’. They needed a more circumscribed model given the specific purpose of making policy measures comparable in terms of their economic growth potential: ‘you just wanted to know if students would flow differently through the educational system as a consequence of such a policy measure’. What that objective in mind, the microeconomists had to bring in a different kind of expertise: ‘In addition, there were two model-builders in this team who made the student flow model. So, you do seek the expertise that you need to bring the project where you want it to be’ (CPB Economist 1). The model-builders came up with a so-called ‘stylized cohort model’ that traced the way students flowed through the stratified Dutch educational system:

And then you can do a small calculation like: This group of people now enters [the school system] and then you can advance for a very long time, you can predict. Because when you implement it, of course, people do not immediately enter the labor market. So it takes a long time, these people have to work for a long time before they achieve it. (CPB economist 3)

The stylized cohort model is based on the structure of the Dutch educational system and on the chances of individuals to flow through that system in a particular way. After completing primary education at age twelve with a particular test score, for instance, a pupil will either go to lower vocational education (four years), higher general secondary education (five years) or pre-university education (six years). Again, after completing lower vocational education, he or she will either go to intermediate vocational education (again four years), higher general secondary education (two years) or enter the labor market at age sixteen. Assuming both the educational structure and the initial distribution of students to be given, an overall rise in student test scores due to performance pay would generate certain shifts in the number of people at each of these levels. Because of their higher grades, some twelve-year olds would now flow to the middle ranks; while some of those already at that level could now enter pre-university education. Overall, students would spend more time in school because higher educational programs take more time to complete (Van Elk et al., 2011a: 18–20).

### Capturing shifts in the quality and quantity of the labor force

**Figure 1.**
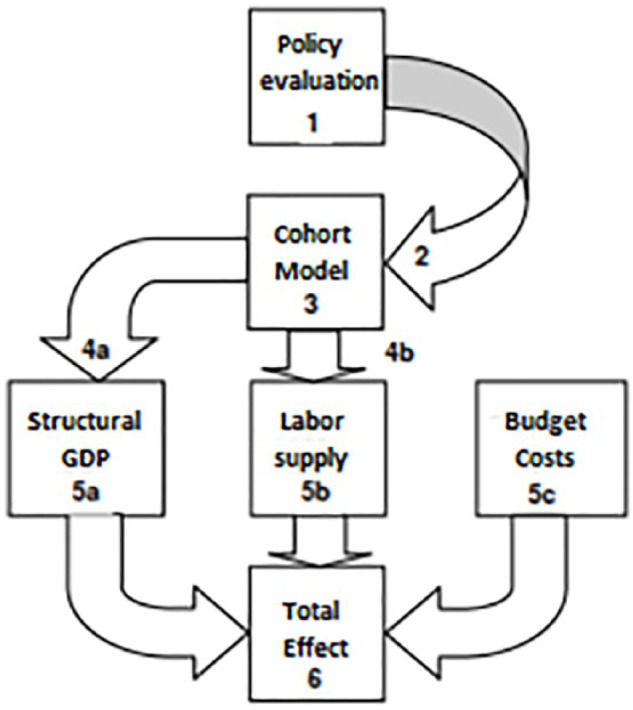
Visualization of the links between experimental studies (‘policy evaluation’) and economic growth (‘total effect’) (Van Elk et al., 2011a: 16).

After they had embedded the stabilized mean effect in the stylized cohort model, CPB economist could tease out the medium term consequences of educational reforms for the student population – not their long-term economic effects. To construct the economic growth potential of policy measures, they had to show what happens when individuals leave the school – i.e. the stylized cohort model – and enter the labor market (see figure 1). Neoclassical growth theorists provided a bridge to the distant economic future. These theories state that the amount of human capital of an individual determines his or her future productivity and income:

One year of education will lead to six to ten percent – some say six to fifteen percent – six to ten percent additional income over the life cycle. So that came from microstudies: so, what someone will earn more from an additional year of education. (CPB economist 2: 4)

**Figure 2.**
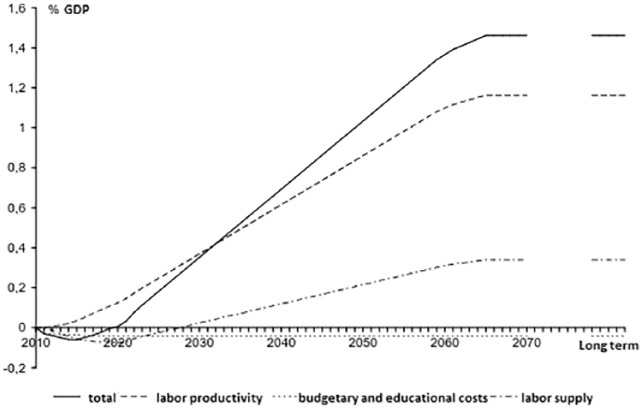
The elaboration of the Dutch scenario for performance pay of the teaching personnel in primary and secondary education. The total GDP effect of performance pay over a sixty-year period relative to basic path is the sum of labor productivity and supply minus the costs (Van Elk et al., 2011a: 65).

An individual with more years of education – a proxy for the slippery notion of human capital – has both a higher chance of participating on the labor market and is also more productive while at work (Van Elk et al., 2011b: 20–21). In terms of participation, increases in educational achievement lead to an increase in the size of the labor force because of the difference in participation rates between the less educated (just above fifty percent) and more educated (close to ninety per cent). A measure like performance pay would first slightly decrease the size of the labor force because individuals spend more time in school. After a while though, there would be a significant increase in size because of the increased participation rate (see ‘labor supply’ in figure 2). The most significant expected increase in GDP, though, comes from a growth model ‘where you look at the amount, the influence of the amount of human capital on growth – these are the so-called endogenous growth models – and you get, they find very high growth figures there’ (CPB economist 3). The key idea of endogenous growth models is that the quality of the labor force – not just its size – would increase because the individuals who benefited from educational reforms would be more productive employees (see ‘labor productivity’ in figure 2). On an aggregate level, an increase in the number of years of education of a whole population will lead to an increase in labor participation and labor productivity on the longer run and thus to a higher GDP level:

Well, you then translate that to 2070. Why that horizon is so distant, is because it is in principle a student population that goes to school now. So, if you are in primary school, then it will be around forty years before the complete labor supply, the whole labor force, is adjusted as a consequence of that policy measure. (CPB economist 1)

After sixty years, the current labor force would be fully replaced and the economy would again be in a steady state (see ‘total’ in figure 2).

### Calculating the optimal budget

After the long-term effects of performance pay on the Dutch economy had been established, CPB economists turned to the budget that was necessary to fully secure its growth potential. Calculating the optimal budget for the full-scale introduction of performance pay was the fourth and final activity in the direction of commensurable futures:

We always say how much you can spend on a measure budget-wise. That partly has to do with … so we have looked a bit at, well, what incentives are offered in these studies and is it ten percent of the salary, is it 50 or 80 or one-and-a-half percent. And we have said on the basis thereof: ‘well this evidence that you see indicates incentives of around, well, x percent. Now, if you take x percent for our population of teachers how much would you have to spend? (CPB economist 1)

On the basis of the size of the bonus in the experimental studies and the number of teachers in the Netherlands, economists from the Bureau calculated in 2010 that a budget of 250 million Euros would be optimal. With a lower budget there would not be enough bonuses to distribute; with a higher budget one would be wasting scarce resources. With the optimal budget, CPB economists were now able to say what the government had to spend to increase the Dutch GDP by one-and-a-half percent in 2070 (CPB and PBL, 2010; 43–44; Van Elk et al., 2011a: 64).

### Constructing commensurable futures

Though this article focuses on performance pay, CPB economists were not interested in individual educational reforms taken separately; rather, they were interested in the overall comparability of policy measures. Their aim was to make the comparison between policies possible by stabilizing a space in which those policies could occur alongside one another:

And in fact, that whole translation with these student flows and the raise you give for additional economic growth that is generated gives you a kind of relative end mark. So, you basically say: ‘This one leads to 1.5 percent more growth and that other measure gives you 0.8 percent. … That is where, that is how you have to actually read it.’ … And we say in principle: ‘What we actually show is the relative… that it has an effect on economic growth and that you can judge policy measures relative to one another.’ (CPB economist 1)

To anchor comparability of policy measures, economists from the Bureau needed to construct the economic growth potential such that the future of each policy would be commensurable with the future of the others. Their newly developed epistemic practice made the long-term consequences of educational reforms sufficiently similar to be grouped together while they remained sufficiently different to be ordered hierarchically (Espeland and Stevens, 1998, 2008). In the hierarchical ordering to which microeconomic forecasting led, performance pay did extremely well. From the range of policy measures that were promising in terms of their potential to increase economic performance, performance pay (1.5%) scored well above additional schooling and coaching of teachers (0.8%), investment in pre-school education (0.5%), the reduction of drop-out levels through labor-intensive coaching (0.2%) and the introduction of monetary incentives for students or the additional time spend on mathematics and language to disadvantaged groups (0.1%). Other measures such as the reduction of class size and increased surveillance by the educational inspection had no economic effect whatsoever. The distance between the two most promising measure became even more pronounced when we take the optimal budgets into account. The second-best option of additional schooling and coaching for teachers would cost the government twice as much – for only half the expected economic effect (Van Elk et al., 2011a: 29–51). In making proposals comparable, microeconomic forecasting served a moral-epistemic ideal too. Due to this new epistemic practice, representatives of political parties and the government could now ‘objectively’ assess the value of each proposal, which enabled them, in turn, to take full responsibility for enhancing the performance of the Dutch economy – be it in the very long run (Espeland and Sauder, 2007; Fourcade, 2016; Sauder and Espeland, 2009).

## The context that is lost in commensuration: Microeconomic forecasting and the fate of epistemic diversity and institutional feasibility

As Espeland and Stevens (1998) point out, the reduction of information that is essential to commensuration has a downside in that it ‘can render some aspects of life invisible or irrelevant’ (p. 314). More directly concerned with the real-world effects of economics, Breslau (2013: 832) demonstrates that the economic framing of markets limits ‘the kinds of justifications that are admissible’ and ‘the kinds of evidence’ that actors can bring to bear on it. Economic policy devices such as microeconomic forecasting not only provide evidence to policy-makers but simultaneously constrain the provision of policy-relevant evidence. Here I draw on the expert judgments of academic economists and civil servants – two key audiences of the Bureau as a boundary organization – to articulate the context that is lost in commensuration. Different actors define that context differently. Academic economists focus in particular on the way CPB economists use experimental studies for forecasting purposes. They emphasize that the extrapolation to long-term economic growth leads to a loss of epistemic diversity. Civil servants from the Ministry of Education, Culture and Science, on the contrary, are less concerned with the intricate details of the economic growth potential of performance pay. Instead, they stress the lack of attention to the institutional feasibility of policy measures in light of the characteristic features of the Dutch educational system.

### ‘You should not throw away information’: Academic economists on the loss of epistemic diversity in microeconomic forecasting

Economists from the Bureau are the first to acknowledge the epistemic uncertainties that come with the establishment of a definite score on economic growth: ‘Can you really say that over, say, 60 years the GDP has increased by one-and-a-half percent? Euhm … then we are the first to say like: ‘these kinds of forecasts are uncertain’’ (CPB economist 1). Academic economists familiar with CPB practices side with that self-assessment: ‘one-and-a-half percent, that will be the case in 60 years; who [inside or outside the Bureau] would attach a kind of certainty to it?’ (Academic economist 1). Epistemic uncertainty, however, is not the main issue that academic economists worry about. They are more concerned about the consequences of microeconomic forecasting for epistemic diversity, i.e. the variety in knowledge claims that is necessary to assess policy measures such as performance pay. For them, epistemic diversity encompasses the full range of available experiments that can be included, the information that makes it possible to contextualize the experimental findings and the non-experimental strands of economics that might be relevant for a complete assessment. At the heart of the matter is a certain paradox concerning how economists from the Bureau deal with economic experiments:

In fact, that is very paradoxical. On the one hand, they say that they only want to use hard evidence for their estimations. Subsequently, to be able to use these estimations requires an enormous amount of tinkering. And all that tinkering is not so hard anymore. (Academic economist 2)

Academic economists point to three specific instances of ‘tinkering’ that lead to the loss of epistemic diversity.

The first instance concerns the choice to include particular studies – and exclude others – to determine the yield of performance pay. That choice, for one, is considered ‘very unclear’ and ‘vague’ and could be the starting point of a long discussion (Academic economist 2). More fundamentally, the Bureau thereby takes a stance in an ongoing debate among experimental economists about ways to deal with the information that experiments contain:

Some people say, well look, we only consider studies with a very good design and we throw away everything we consider to be ill-designed. But you can also say that all studies provide information that you would like to include in your assessment. … Then you have to ask yourself: Is an American study with a solid design more informative then a study – perhaps a good study but with a somewhat less solid design – that is conducted in the Netherlands? I myself think: You should not throw away information. … you still have to look whether there is information in it. (Academic economist 3)

The tendency of CPB economists to opt for (foreign) studies with a stronger design at the cost of (Dutch) studies with a weaker one might come at the price of already losing much information in the selection process.

The second instance of tinkering is linked to the calculation of the average effect of performance pay on student test scores. Here, the sheer diversity in the experimental results – some positive, some negative – is the issue at stake:

Interviewer: *What I wonder about is, … how far can you strike an average on the basis of ambivalent results?*

Interviewee: Well that is indeed difficult. Especially when the results are so diverse. There are of course so many degrees of freedom where that diversity can be attributed to: to the design, to the context and so forth. Look, if it was all unambiguous, if it would follow from all studies that it works: fine. Then you say that it works regardless of the context. But if you find many different things then it is very difficult to draw conclusions from them because one can attribute these differences to many factors. (Academic economist 3)

The combination of positive and negative experimental findings about the effects of performance pay leads to the question which factors might offer an explanation for the diversity. By striking an average on the basis of diverse results one can no longer see that there was diversity but you equally lose track of the contextual factors that contributed to that diversity in the first place.

The third and final instance of tinkering has to do with the translation of foreign experiments to Dutch education. Economists from the Bureau admit that there are no standards on how to translate experimental results from one educational context to the other:

Do you have the exact justification why it should be half of it? No. … And then you say at a particular moment, okay, the question is: Do we get the same effect? Then we show at a particular moment: suppose it is decreased by half. So, there is no exact justification why it should be half or three quarters. That’s not there at that moment. (CPB economist 1)

Academic economists similarly point to the lack of justification for decreasing the average effect, saying that ‘there is no experimental evidence that you have to divide that in two; it could have been one-third or two-thirds’ (Academic economist 2). But because CPB economists ‘partly reduce the value of it being experimental’ by such a decrease, they should also be more open than they currently are to non-experimental studies: ‘And then you can also say: That cross-sectional research conducted in the east of the Netherlands becomes valuable too. It is not perfect either, so you get different imperfect sources’ (Academic economist 2). It becomes more difficult to exclude other sources of knowledge once you start tinkering with the experimental results in order to construct policy-relevant evidence.

### ‘School directorates have the highest degree of autonomy in Europe and the whole world’: Civil servants on the institutional feasibility of performance pay in the Netherlands

Civil servants from the Ministry of Education, Culture and Science were not fully convinced that performance pay would work, and thought that the Dutch government started with a ‘somewhat inflated expectation of the effectiveness of this policy measure’ (Civil servant 2). Part of their reluctance had to do with the fact that the economic experiments – from which CPB economists extrapolated to long-term economic effects – were themselves often short-lived:

But we were somewhat skeptical in general, to say the least, about the effectiveness of performance pay. In itself, the CPB had demonstrated that there were effects but nobody was able to demonstrate whether they were durable because the experiments often lasted only for one or two years. (Civil servant 2)

Although worried about the lack of durable effects in the experimental literature, the absence of a well-developed political agenda for the implementation of performance pay was a bigger and more immediate concern for civil servants. The optimal budget for performance pay reappeared in the coalition agreement and the economic growth potential of educational reforms reappeared in political justifications for the measure – but so, eventually, did the question how to actually spent a quarter of a billion euros on wage incentive schemes for teachers.

Focusing on the economic growth potential and optimal budget of policy measures, the Bureau had never considered the question of their feasibility in light of the institutional characteristics of Dutch education. As the previous section demonstrates, CPB economists did take the Dutch educational context into account on occasion but only in so insofar as they needed to decide about diminishing the effects found in foreign experimental studies and had to calculate the optimal budget for a full-scale introduction of the policy measure. As economic experts, the reception of educational reforms like performance pay by teachers and school directorates was not a core concern to them: ‘whether we are all really preoccupied with the question of whether or not it will be well received? That is not up to us’ (CPB economist 1). In similar vein, the Bureau was not preoccupied with the way an educational reform should be implemented. In the CPB advisory reports on promising policy measures in education ‘there is no story about implementation. That is not something that is really taken into consideration. Perhaps that is a lacuna’ (CPB economist 3). When asked for the institutional possibilities for performance pay in the Netherlands, CPB economists point to the government’s ability to centrally orchestrate its introduction via ‘the usual collective labor agreement on the national level … many policy measures are implemented at the national level so there are indeed possibilities for a government to do it this way’ (CPB economist 1).

From the start, civil servants had qualms about the feasibility of a centrally orchestrated introduction of performance pay in the Dutch educational context. The main issue at stake, for them, was that the government had little to say about the ways in which schools actually spend allotted budgets. The reason for this lack of power over the spending behavior of educational institutions had to do with broader deregulation movement, starting in the mid-1980s, that departed from a cost-claiming system that severely constrained schools in their financial decisions (Karsten and Meijer, 1999: 421–426). The lump sum financing that eventually emerged from this political shift gave school directorates a lot of discretionary power in financial matters. This makes Dutch school management stand out vis-à-vis the management of schools in other countries:

Look, the Netherlands is a country where school directorates have the highest degree of autonomy in Europe and the whole world. There is no [other] country in which the government gives huge sums of money to a private corporation, supervises a little and establishes some goals and gives a couple of centrally standardized tests, and for the rest: good luck with it. (Civil servant 1)

In light of that political-historical trajectory, the employers’ council that represents the interests of the school directorates at the ministry was not particularly favorable to the measure: ‘and to be honest, employers were thinking a bit on along these lines: “government, are you really going to interfere with the way I reward my personnel? Shall we just leave that up to me?”‘ (Civil servant 1).^6^

In addition to their initial skepticism about the durability of the experiments, the civil servants started with an overall lack of faith in the grand scale of the whole operation: ‘I don’t think we ever believed that we could have spent that 250 million euros on it’ (Civil servant 2). With such a large budget in mind, the government would have to turn to the unusual measure of funding schools for a very specific purpose:

But we never came much further than to identify it as a problem, as a question. Never came much further with this state secretary, too. So, the only thing we could think of at the time was to earmark the money. Then you have to say: ‘you will receive this as a directorate and we assume … this is not part of the lump-sum budget, this is part of a direct form of financing for this particular purpose’. Well, to be honest, that is also in the Dutch context almost undoable. (Civil servant 1)

Tasked with the implementation of performance pay, whatever the practical difficulties, civil servants came up with a proposal that made it possible to postpone the problem of the full-scale introduction of performance pay. They pushed the state secretary to start with a series of experiments in which schools could participate until the measure would (somehow) be fully implemented in a later stage. Because school directorates could apply on an individual and voluntary basis, the government side-stepped the collective labor agreement that would otherwise have been necessary: ‘that was an additional reason that experiments came in handy. According to me, there was no solution at all to this problem, how to do that’ (Civil servant 2). In the end, the problem resolved itself. With significant efforts – by actively searching for school directorates who would be willing to voluntarily apply for funding – civil servants managed to find recipients for the first ten million of the experimental budget. But even that first round of experiments failed to materialize. As a result of an aggressive union campaign for the cancellation of performance pay, and the overall lack of enthusiasm of the employers’ council, the Dutch government decided to terminate the measure in 2012 (aob, 2011, 2012).

### The context that is lost in commensuration

The political dynamics that led to the failure of performance pay takes us well beyond the confines of the article. However, the rapid downfall in the preliminary stage of the experimental process does allow us to qualify the evidence that led to the predominantly positive expectations about performance pay among economic experts and politicians. That qualification is relevant because policymakers often need different kinds of evidence to address different social and political concerns (Parkhurst, 2017). Drawing on the perspectives of academic economists and civil servants, this section revealed the context that was lost in commensuration. The endeavor to construct commensurable futures first led to a loss of epistemic diversity. A single score for economic growth makes the ambiguity in the experimental results, the experimental and educational context required to make sense of these results and the non-experimental studies in the economics of education invisible – and hence irrelevant for the assessment of policy measures. Given the ‘tinkering’ that was necessary to turn the experimental studies into policy-relevant evidence, academic economists wonder whether that process of commensuration was warranted. At a minimum, this led them to the conclusion that ‘if you start refining some experiments then you must accept that non-experimental research is also informative for your decision’ (Academic economist 2). Some go further and say that the extrapolation to long term economic growth is ‘predominantly guesswork’ and ‘for show’ in the sense that ‘you think you can make nice headlines with it’ (Academic economist 3).

Starting from their concerns about the feasibility of performance pay in the Dutch educational context, civil servants had to find a way to deal with the high expectations among economic experts and conservative-liberal politicians about its effectiveness. Their concern for the institutional feasibility of performance provides a second angle on microeconomic forecasting: The comparison of policy measures in terms of their economic growth potential reduced the visibility of the legal and institutional rearrangements that would be necessary to make them work. The exclusive focus on the economic growth potential and optimal budgets led CPB economists to bracket all questions related to the feasibility of spending a quarter billion Euros on performance pay. Again, the potential problems with its reception and implementation where all hidden behind a single figure of long-term economic growth.

## Conclusion

In this article, I have analyzed how a team of economists at the Netherlands Bureau for Economic Policy Analysis created a new forecasting practice that made the distant economic future part of the political present. Three questions stood out. The first part of the article was concerned with the question of the institutional preconditions for microeconomic forecasting. The Bureau, as a boundary organization, turned to microeconomics to remain policy-relevant in a dynamic political environment and embraced the criterion of solid experimental support in response to developments in academic economics. The second part of the article addressed the question of how to understand microeconomic forecasting as a particular practice of producing knowledge about the economic future. I showed that the endeavor to compare policy measures on the basis of their economic growth potential was conditional on a process of commensuration in which the future consequences of these measures became sufficiently similar to allow for comparison and remained sufficiently different to establish a hierarchy between them. The third part of the article addressed the question of how far microeconomic forecasting not just enabled but simultaneously constrained the provision of policy-relevant evidence. Focusing on the context that was lost in commensuration, I argued that microeconomic forecasting did not allow for much reflection on the epistemic diversity within economics and on the institutional feasibility of substantial educational reforms in the Dutch educational context – evidence that might be of significant importance for a balanced assessment of a policy measure.

Beyond the specificities of the case, the article has a wider relevance for the study of the economics-infused sociotechnical tools that policymakers use to see the world and make decisions about it. More specifically, it outlines how to study microeconomic policy devices from a sociological perspective against the background of the market bias in STS research on economics (Hirschman and Berman, 2014). In doing so, my analysis makes it possible to include a new range of epistemic practices that are currently quite marginal to STS research. These epistemic practices range from the economic theories and models of agency, information and incentives that are used to articulate problems and solutions in the public sector to the increased role of behavioral economists in policymaking processes (Beckert, 2016: 245–268; Bowles, 2016; Thaler and Sunstein, 2008). STS research could contribute to the study of such practices through detailed historical and sociological analyses of their epistemic characteristics. At the same time, the very status of these epistemic practices as policy devices requires STS researchers to cast their net a bit wider. With regard to the institutional preconditions for the political authority of the ‘fifth branch’, such a wider perspective would entail an analysis of the position of economic experts as legitimate speakers on matters of public policy and the internal and external dynamics that lead them to take new stances within the political field (Bourdieu, 1991; Jasanoff, 1990). With regard to the political consequences of economic policy devices, such a wider perspective would encompass the circulation of economic evidence beyond the initial context of the epistemic practice in question. The analysis of circulating evidence would lead us to the (dis)trust of political actors in – and strategic responses to – economic policy devices as well as to the controversies that might follow from the confrontation with the ‘lay’ perspectives of practitioners and organized interests (Nelkin, 1984; Porter, 1995; Wynne, 1996).

## Notes

1. CPB documents are publicly accessible but the Bureau itself was not particularly prone to provide access to multiple experts on microeconomic forecasting. When I targeted multiple economists at the same time, access was restricted to one economist. To broaden the evidential base, I targeted former CPB economists who had worked on microeconomic forecasting or on other projects in the economics of the public sector. Academic economists and civil servants were generally willing to speak about the failed policy experiment with performance pay and the possibilities and constraints of the bureau’s new epistemic practice (one economist declined because (s)he could not see the benefit of spending time on my project).

2. The bureau’s political success in the decades after its establishment led to the creation of other future-oriented institutions such as the Scientific Council for Government Policy in 1972 and the Social and Cultural Planning Bureau in 1973. Although these planning bureaus were modeled on the CPB, they were also created as alternative sources of expertise for different ministries to counter the dominance of the CPB’s model-based practices. Though partly countervailing powers, the planning bureaus refrain from open competition with each other and instead choose to informally coordinate their actions and negotiate the relations between them (Halffman, 2009; Halffman and Hoppe, 2005).

3. On other occasions, the bureau expressed a keen awareness that being attuned to the needs of policy-makers can never be taken for granted and comes with the need to be flexible: ‘policy relevance is no static given. The world changes and the needs of our clients change with it. As such, it is important to adapt to these changing needs in the research agenda’ (CPB, 2007: 6).

4. Some CPB economists lament that the exclusive focus on experimental studies displaces other strands of economics: ‘people say: experiments, experiments are important because, for the rest, all other knowledge is useless or something. That is the impression I have.’ On the downside, the new ‘engineer-type of approach’ led to the exclusion of ‘a somewhat bigger vision than just the pinpricks of these interventions [ticks with finger on the surface of the table], a somewhat broader vision’ (CPB economist 4).

5. The actual statement was made by the Jacques Schraven, chairman of the employers’ organization, at a meeting of the Social Economic Council on March 22 2002: ‘If the CPB had been charged with the evaluation of (the possible consequences of) the invasion of Normandy, those ships would still be floating out by the coast’ (quoted in CPB, 2003: 13).

6. Academic economists with a keen eye for the Dutch educational context equally consider the central steering of educational reforms like the one considered here to be ‘unrealistic’ and ‘impossible in practice’ because ‘the ministry cannot appoint schools and say to them: you have to introduce performance pay’ (Academic economist 3).
